# Carbon nanotubes as electrophysiological building blocks for a bioactive cell scaffold through biological assembly to induce osteogenesis

**DOI:** 10.1039/c9ra00370c

**Published:** 2019-04-16

**Authors:** Saibo Qian, Zhilin Yan, Yongjie Xu, Huaping Tan, Yong Chen, Zhonghua Ling, Xiaohong Niu

**Affiliations:** School of Materials Science and Engineering, Nanjing University of Science and Technology Nanjing 210094 China hptan@njust.edu.cn; Department of Orthopaedics, Jinling Hospital Nanjing 210002 China; Department of Luoli, Nanjing Hospital of Integrated Traditional Chinese and Western Medicine Nanjing 210014 China 781970731@qq.com.cn

## Abstract

Bio-functional cell scaffolds have great potential in the field of tissue regenerative medicine. In this work, a carbon nanotube (CNT) gel scaffold *via* specific pairing of functionalized nucleobases was developed for specifically targeted drug delivery and *in vitro* osteogenesis. The CNT gel scaffold with nano-fibrous architectures was established by Watson–Crick base pairing between thymine and adenine of low molecular weight heparin, respectively. As scaffold precursors, adenine and thymine functionalized heparin derivatives could additionally bind cell growth factors by the affinity interaction. The resulting nano-fibrous gel scaffolds showed excellent mechanical integrity and advanced electro-physiological functions. Potential application of the electrophysiological CNT gel scaffold in bone tissue engineering was confirmed by encapsulation of human adipose-derived stem cells (ASCs). Our results indicate that the electrically conductive networks formed by CNTs within the nano-fibrous framework are the key characteristics of cell scaffolds leading to improved ASC organization and differentiation by an extra electrical stimulus (ES). Specifically, ASCs cultured in bio-electrical gel scaffolds showed ∼4 times higher spontaneous osteogenesis in combination with bone morphogenetic protein 2 (BMP-2), compared to those cultured on pristine hydrogels. This electrophysiological CNT gel scaffold containing BMP-2 exhibited beneficial effects on ASC activity and osteogenetic differentiation, which suggested a promising future for local treatment of bone regeneration.

## Abstract 2

Bio-functional cell scaffolds have great potential in the field of tissue regenerative medicine.

## Graphical abstract

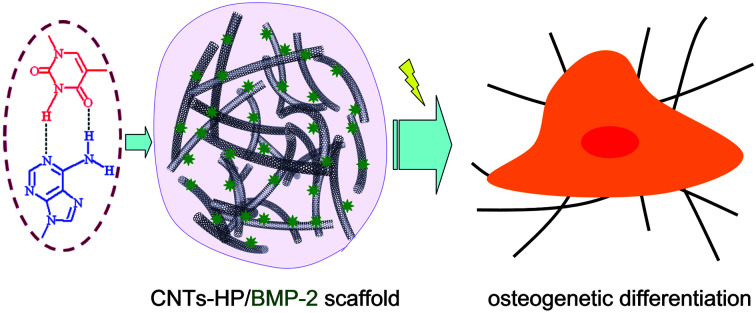

## Introduction

Injectable cell scaffolds have great potential in the field of tissue regenerative medicine, and are widely used as cell carriers and artificial tissues.^1^ Biodegradable hydrogels have served as highly functional cell scaffolds in addition to cell growth factors (GFs), which allow easy and homogeneous cell distribution within any defect size or shape. Although the versatility of cell scaffolds has been broadly exploited, nano-structured and bio-functional gel scaffolds are limited by conventional biopolymers and methods. There is significant interest in exploiting cell scaffolds with nano-structured architectures to construct mimics of the extracellular matrix (ECM) for cell-culture applications. In bone tissue engineering, a major issue in the use of gel scaffolds is that the establishment of a biomimetic environment is necessary for *in situ* osteogenesis of adipose-derived stem cells (ASCs).^2^

Clinically, ASCs have demonstrated a broad *in vitro* capacity for differentiating toward cell types of all 3 germ layers, which suggests that they have wide utility in a range of regenerative strategies for multiple tissue types. Recently, undifferentiated ASCs have been seeded on a variety of cell scaffolds and examined *in vivo* for bone regeneration.^3^ ASCs are similar to bone marrow-derived stem cells (BMSCs) in that they are capable of maturing toward multiple mesodermal tissue types, including bone, cartilage and adipose; they are immunosuppressive, and they show similar surface protein marker expression. Importantly, ASCs are unique from BMSCs because they can be obtained easily in a minimally invasive manner from adipose tissue harvested through lipoplasty or liposuction in clinically applicable numbers without the need of expansion in culture. Furthermore, ASCs senesce is later than BMSCs, which may be beneficial for treating chronic or persistent conditions. For these reasons, ASCs are appealing for cell-based therapies in bone repair and regeneration.

The synthesis of a new nano-structured and bio-functional gel scaffold might therefore permit manipulation of inductive properties for osteogenesis differentiation of ASCs. Carbon nano-tubes (CNTs) have emerged as a promising nano-structured material for biomedical applications due to their unique atomic configuration, optical, mechanical and electronic properties, high surface area to volume ratios, as well as variable fictionalizations.^4^ Properly functionalized CNTs are biocompatible, non-immunogenic and photo-luminescent. These properties show potential for CNTs to be clinical nano-carriers for drug delivery and imaging. Therefore, incorporation of CNTs into biopolymer hydrogel, and potentially other biomaterials, could be useful in creating multifunctional scaffolds for both therapeutic purposes and *in vitro* studies. As for osteogenesis scaffolds in bone tissue engineering, most of the previous methods in the literature describing the novel functionalities are present only on the toughening modification of the CNTs for gel substrate, rather than the bio-electrical conductivity on stem cells.^5^

Bone formation occurs normally under the control of cells and a variety of interacting growth factors in the microenvironment.^6^ There is evidence that the ability of ASCs to grow and differentiate varies among physical structures and changes with bioactivities of scaffolds. Defining these variations in molecular mechanisms of osteogenesis will facilitate the development of ASCs-based therapies. Herein, we hypothesized that through hybridization, the nano-structured and bio-electrical gel scaffold can provide biochemical cues to the osteoid-like gels to generate an artificial niche for osteogenesis differentiation. It is a difficult challenge to create a bio-functional cell scaffold from ASCs to *in vitro* build an organized cellular construct that would permanently stimulate osteogenesis differentiation and native bone regeneration. Besides of GFs, we sought to further stimulate ASCs in CNTs-based cell scaffold by using home-designed bio-electric, in order to promote osteogenesis differentiation. Different from the existing cell scaffold technologies, we pioneered a bio-electrical cell-affinity stimulus, by which scaffold was utilized to induce osteogenesis of ASCs. Our group has employed biological assembly through specific nucleobase pairing to assemble into biodegradable and biocompatible hydrogels for GFs delivery.^7^ We have demonstrated that biopolymers modified with thymine and adenine functionalities spontaneously form hydrogel scaffolds *via* the Watson–Crick base pairing that support GFs delivery and cell encapsulation. These successful results clearly illustrate that specific nucleobase pairing promise as cell scaffolding biomaterials for tissue engineering and regenerative medicine. In this work, we present this flexible way to further assemble an electrophysiological CNTs scaffold with nano-fibrous architectures that are capable of inducing osteogenesis of ASCs. Our strategy is to use adenine and thymine functionalized heparin as hydrophilic and flexible bridges, which could synchronously bind bone morphogenetic protein (BMP-2) by specific affinity interaction.

## Experimental section

### Materials

Adenine, thymine, sodium heparin (*M*_w_ 18 kDa), 1,9-dimethylmethylene blue and 3-(4,5-dimethyl-thiazol-2-yl)-2,5-diphenyltetrazolium bromide (MTT) were purchased from Sigma-Aldrich. Chemical vapor deposition grown single-walled carbon nano-tubes (CNTs) were supplied by Thomas Swan & Co. Ltd (Elicarb®). *N*-Hydroxysulfosuccinimide (sulfo-NHS) was purchased from Pierce Biotechnology Inc. (Rockford, IL). CyQuant Cell Proliferation Assay Kit, trypsin–EDTA solution, streptomycin and penicillin were purchased from Invitrogen Co. (Carlsbad, CA). Recombinant human BMP-2 (rhBMP-2) and the BMP-2 Quantikine ELISA Kit were obtained from R&D Systems (Minneapolis, USA). All other chemicals were used as received.

### Synthesis of nucleotide modified biopolymers

#### HP-adenine

Diethyl ether (0.07 mmol) and CHCl_3_ (0.035 mmol) was added to phosphorus pentoxide (0.035 mmol) with stirring and the mixture was heated under reflux for 12 h at 50 °C to get a clear solution. The solvent was then distilled off under vacuum to obtain polyphosphate ester. Adenine (0.004 mmol) was dissolved in 50 mL of dimethylformamide with the addition of 0.25 mL of concentrated HCl with magnetic stirring. Polyphosphate ester was added to heparin (0.0006 mmol) in 30 mL of dimethylformamide, followed by dropwise addition of adenine solution, and the mixture was heated to 50 °C for 24 h. The reaction was cooled at RT and dimethylformamide was distilled off *in vacuo*. The moist residue was dissolved in water and kept in an ice-box for about 1 h to precipitate unreacted adenine. To the clean aqueous layer, ammonia was added to bring pH to 10.0. The ammonia layer was extracted with ethyl acetate and the organic layer was evaporated. The product was dissolved in water and further purified by dialysis (MWCO 3500) against ultrapure water for 3 days. The purified solution was lyophilized to give solid foam.

#### HP-thymine

The clear solution of thymine (0.004 mmol) was obtained by dissolving in 25 mL of H_2_O with the addition of 0.25 mL of concentrated HCl solution. Polyphosphate ester was added to heparin (0.0008 mmol) in 50 mL of H_2_O, followed by dropwise addition of thymine, and the mixture was heated to 50 °C for 20 h and cooled under ice for about 1 h to precipitate unreacted thymine. The acid layer is neutralized by the addition of ammonia. The ammonia layer was extracted with ethyl acetate and the organic layer is evaporated. The product was dissolved in water and further purified by dialysis (MWCO 3500) against ultrapure water for 3 days. The purified solution was lyophilized to give solid foam. For brevity, the adenine and thymine functionalized heparin are designated as HP-A and HP-T, respectively.

### Preparation of scaffold precursors

Amine-functionalized CNTs (CNT-NH_2_) were synthesized according to an already reported procedure slightly modified.^7^ The loading of free amines determined by colorimetric Kaiser test was 90 μmol g^−1^, corresponding to 110 μmol g^−1^ of carbon taking into account the filling yield. The CNT-NH_2_ was also characterized by TGA performed this time under inert atmosphere, confirming the occurrence of the functionalization. Scaffold precursors were synthesized by grafting HP-A and HP-T onto CNT-NH_2_, which were referred as CNT-HP-A and CNT-HP-T, respectively. To graft the nucleotide modified biopolymers onto CNT-NH_2_, 0.5 g of HP-A and HP-T was dissolved in 200 mL nanopure H_2_O and incubated with EDC at 4 °C for 48 h, respectively. CNT-NH_2_ was dissolved in nanopure H_2_O and added into the nucleotide modified heparin solutions under agitation with a final pH value of 5.6. The mixture was incubated at room temperature for 24 h before dialysis (MWCO 5000) for 3 days. To verify the structure of CNT-HP-A and CNT-HP-T, ^1^H NMR spectra were measured at ambient temperature using D_2_O as a solvent.

### Assembly of scaffolds

CNT-HP-A and CNT-HP-T were dissolved in phosphate buffered saline (PBS) to form precursor solutions at a concentration of 0.5–3.0 wt%, respectively. For the assembly of scaffold, solutions of CNT-HP-A and CNT-HP-T were thoroughly mixed with 1 : 1 ratio at room temperature by vigorous pipetting. For preparation of GFs loaded gels, BMP-2 was dissolved in the CNT-HP-A and CNT-HP-T solution in an ice bath before mixing. The final concentration of BMP-2 was fixed as 50 ng mL^−1^ (CNTs/BMP-2). To make control group, the same concentration of HP-A and HP-T in PBS was prepared. The HP-A and HP-T solutions with or without BMP-2 were thoroughly mixed by pipetting.

### Chemical structures

Fourier transformed infrared (FT-IR) spectra of nucleotide functionalities of the HP-A and HP-T were measured to confirm the chemical composition. The obtained samples were recorded with FT-IR spectrometer (Nicolet Avatar 360, USA) against a blank KBr pellet background. The nucleotide functionalities of the HP-A and HP-T were further characterized *via*^1^H NMR (Bruker Avance). ^1^H NMR spectroscopy results indicated that the nucleotide functionalities on HP-A and HP-T were estimated to be 57.4% and 62.3%, respectively.

The grafted contents of HP-A and HP-T in the CNTs were determined quantitatively using a 1,9-dimethylmethylene blue method.^8^ The CNT-HP-A and CNT-HP-T were freeze-dried and digested with papain in buffer of 0.1 M KH_2_PO_4_, 5 mM Na_2_EDTA and 5 mM cysteine·HCl at pH 6.0 and 60 °C for 6 h. The dye solution was prepared by dissolving 16 mg of 1,9-dimethylmethylene blue in 1 L distilled water containing 3.04 g glycine, 2.37 g NaCl and 95 mL 0.1 M HCl. 2 mL 1,9-dimethylmethylene blue solution was added into 100 μL of the papain digested solution after filtration. After 5 min, the absorbance was measured at 525 nm by UV-vis spectroscopy (UV-Probe 2550, Shimadzu).

### Characterization of scaffolds

#### Rheological experiment

Gelation kinetics of the gel scaffolds were evaluated using a commercial rheometer (AR2000, TA Instruments, New Castle, USA) with the parallel plate geometry (25 mm in diameter). The reaction mixture was quickly mixed and poured onto the center of the bottom plate. Standard low viscosity oil was spread at the side of the geometry to prevent water evaporation during the measurements. The evolution of storage (*G*′) and loss (*G*′′) moduli at a constant frequency of 0.5 Hz and constant strain of 0.05 was recorded as a function of time. The gelation time, as a kinetic parameter for network formation, was defined as the time of crossover of *G*′ and *G*′′.

#### Morphologies

Morphologies of scaffolds were characterized by utilizing SEM. For morphologies observation, the scaffolds were critical point dried (K850, Quorum, UK), and the cross-sectional morphologies were viewed using a SEM instrument (JEOL, JSM-6700F) operated at 5 kV accelerating.

#### Weight loss

Weight loss of initially weighed scaffolds (*W*_0_) was monitored as a function of incubation time in PBS at 37 °C. At specified time intervals, scaffolds were removed from the PBS and weighed (*W*_t_). The weight loss ratio was defined as 100% × (*W*_0_ − *W*_t_)/*W*_0_. The weight remaining ratio was defined as 1–100% × (*W*_0_ − *W*_t_)/*W*_0_.

#### Compressive modulus

For compressive modulus experiment, solutions described above were injected into a 24-well culture plate for mixing to obtain columned scaffolds. Compressive modulus of elasticity was measured in the elastic region of scaffolds using a dynamic mechanical analyzer (DMA-7, PerkinElmer) in unconfined compression at a constant stress rate of 40 mN min^−1^ up to 20% strain at 37 °C.

#### Electrical characterization

The current–voltage (*I*–*V*) curves were obtained at ambient temperature using a two-probe station (H19S00556, HiSOL, Japan). The currents were recorded by sweeping the voltage from 0 to 5 V. The cables of the probe-station were connected to the inter-digitated electrodes to obtain reproducible and accurate conductivity measurements. The impedance values were measured using a CompactStat Potentiostat (CompactStat, Ivium Technologies, Netherlands) with IviumSoft software. The frequency was altered from 0.2 to 5 Hz, and the perturbation amplitude was 25 mV.

#### Drug release

For BMP-2 release experiment, 100 μL of scaffolds were suspended in 1 mL of PBS at 37 °C, provided a reservoir into which BMP-2 could be released from the gel complex and subsequently measured during culture. At predetermined intervals, a 200 μL sample of release medium was extracted from the sample vials and replenished with 200 μL fresh release medium to maintain a constant volume. Samples were centrifuged and supernatants were collected and stored at −20 °C until analysis. Samples were refreshed with new PBS and vortexed after each collection. The release of BMP-2 was determined by the ELISA kit assays.

### Cell culture

Human adipose-derived stem cells (ASCs) were isolated from human adipose tissue obtained from elective cosmetic surgery procedures under the institutional guideline.^9^ The fat tissues were minced with scissors in the collagenase solution consisted of Hanks' balanced salt solution (3.0 mL g^−1^ of fat) (Sigma-Aldrich, St. Louis, MO), bovine serum albumin (fatty acid free, pH 7.0, 3.5 g/100 mL Hanks') (Intergen Company, Purchase, NY) and 1% type II collagenase (3.0 mg g^−1^ of fat) (Worthington Biochemical Corporation, Lakewood, NJ). The centrifuge tubes were shaken at 100 rpm for 50 min at 37 °C. Following digestion, the content of each tube was filtered through double-layered sterile gauze. The filtrates were then centrifuged at 1000 rpm for 10 min at 37 °C, and a three layer suspension, consisting of a fatty layer on the top, a serum layer in the middle, and a cellular pellet at the bottom of each tube, was obtained. The fatty layer and most of the supernatant was aspirated off, leaving the pellet intact at the bottom. The pellet in each tube was then suspended in 10 mL of erythrocyte lysis buffer (pH 7.4), vortexed, and centrifuged again at 1000 rpm for 10 min at 37 °C. The pellets were suspended in the plating medium consisted of DMEM/F12 with 10% heat-inactivated fetal bovine serum, 1% penicillin/streptomycin and 1% Fungizone (all products obtained from Gibco, Invitrogen Corporation, Carlsbad, CA). Adherent ASCs were expanded for a period of 5–8 days at 37 °C, and the medium was changed every other day until the cells achieved 80% confluence.

Cell experiments were carried out in sterilized gel scaffolds with an extra electrically stimulus (ES). ASCs were plated at a density of 5 × 10^6^ cells per mL in 24-well plates on samples, differentiated in 24 well inserts 0.4 μm pore size. The ASCs cultured on the CNT gels were electrically stimulated at day 2 of culture using the inter-digitated electrodes under the scaffolds. The ES was applied using a waveform generator (Hioki 7075, Hioki, Japan) under a specific program (frequency 1 Hz, voltage 3 V, duration 10 ms) for three continuous days. The electrically stimulated time of the ASCs on the scaffolds was 30 min in 24 h. The medium was replenished every day during the stimulation of the scaffolds to eliminate any side effect of the generated charge. The number of ASCs encapsulated in scaffolds was quantified using the CyQuant Cell Proliferation assay. Cells were fixed and critical point dried for SEM to observe cell morphology in samples. Cytotoxicity on scaffolds was evaluated by WST1 reagent test. Cell activity and differentiation was evaluated at the end of experimental times in cell culture supernatant by Immunoenzymatic Assay kits (Invitrogen, USA) according to the manufacturer's instructions for ALP, COL-1, OCN and TGF-*β*1. The cells-scaffold construct were cultured in 96 well plates in complete media and on day 7 and 14 samples were washed with PBS followed by addition of 50 μL lysis solutions (0.5% TritonX-100) in each well. Thereafter, 200 μL of 1 mg mL^−1^ reaction substrate was added to each wells and incubated for 1.5 h at 37 °C following the manufacturer protocol. Absorbance was recorded at 405 nm using a spectrophotometer (ELx800, BioTek Instruments, Inc., USA). Gene expression of ALP, COL-1, TGF-*β*1 and OSX were also evaluated by quantitative polymerase chain reaction (qPCR). After culturing in osteogenic induction media for 14 d, ASCs under different scaffolds were treated with TRIZOL Reagent (Invitrogen) to extract the total RNA. The RNA concentration, purity and integrity were performed using a Q-5000 spectrophotometer (Quwell) at 260/280 nm and agarose gel electrophoresis. For cDNA synthesis, mRNA was reverse-transcribed into cDNA using the 5× PrimeScript RT Master Mix (TaKaRa) at 37 °C for 15 min and 85 °C for 5 s according to the manufacturer's protocol. The synthesized cDNA samples were subjected to determine the expression of ALP, COL-1, TGF-*β*1 and OSX. Gene expression was quantified by Real-Time qPCR using SYBR Green master rox (Roche Diagnostics Ltd, Mannheim, Germany) with a 7500 ABI Real-Time PCR System (Applied Biosystems, Foster City, CA, USA). The housekeeping gene was glyceraldehyde 3-phosphate dehydrogenase (GAPDH).

### Statistical analysis

The experimental data were reported as mean ± standard deviation. Statistical analysis was performed using the two population Student's *t*-test. Statistical significance was set to *p* value ≤ 0.05.

This study was performed in strict accordance with the NJUST guidelines for the care and use of laboratory animals and was approved by the Institutional Animal Care and Use Committee of Nanjing University of Science and Technology (Nanjing, China).

## Results and discussion

### Scaffold formation

**Fig. 1:**
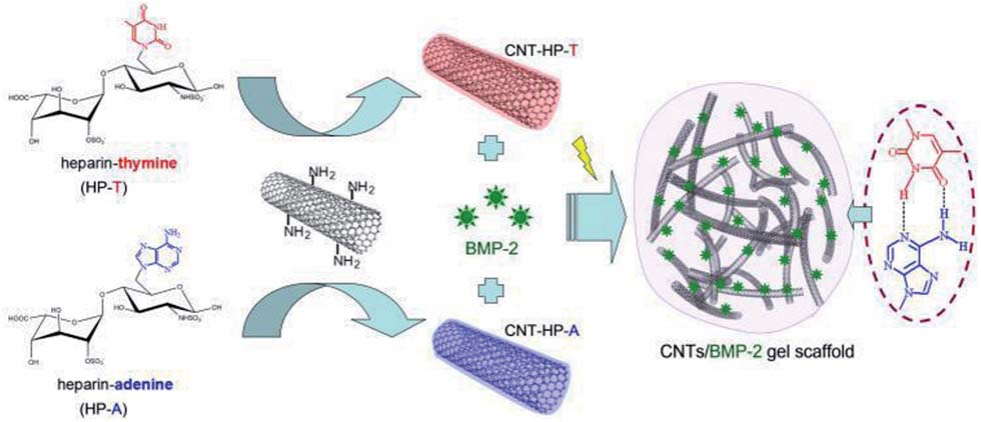
Schematic assembly of CNT gel scaffold through the Watson–Crick base pairing. The scaffold precursors were designed with adenine and thymine functionalized heparin on CNTs backbone respectively, which are accessible for conjugating *via* specific nucleobase pairing in aqueous environment. Heparin was able to provide stabilization of BMP-2 activity and prolonged delivery *via* affinity interaction.

The bio-electrical CNT gel scaffold is established by the Watson–Crick base pairing between thymine (T) and adenine (A) *via* the hydrogen bonding (Fig. 1). For nucleobase pairing, heparin was functionalized with adenine and thymine functionalities (referred as HP-A and HP-T), respectively. HP-A and HP-T were further grafted onto the aminated CNTs as assembly precursors, respectively (referred as CNT-HP-A and CNT-HP-T). After dissolution and mixture of CNT-HP-A and CNT-HP-T in an aqueous environment, a nucleobase paired CNT gel network was assembled due to the formation of pairing complexes. Heparin is a negatively charged glycosaminoglycan (GAG), which is composed of repeated disaccharide units of alternating glucosamine and glucuronic residues heterogeneously modified by carboxyl groups and *N*- or *O*-linked sulfate. Heparin has been clinically used as an anticoagulant agent. The ability of heparin to sequester and stabilize GFs due to specific affinity interaction has been exploited in the production of cell scaffolds that can mediate cell proliferation and differentiation.^10^ The heparin in cell scaffold should be able to provide stabilization of GFs activity and prolonged delivery during bone regeneration. To investigate the utility and versatility of the CNT gel scaffolds for GFs delivery, the bone morphogenetic protein (BMP-2) was employed as the model for affinity delivery. The BMP-2 is easily accessible to heparin, thus enabling affinity interactions in aqueous environment.

**Fig. 2:**
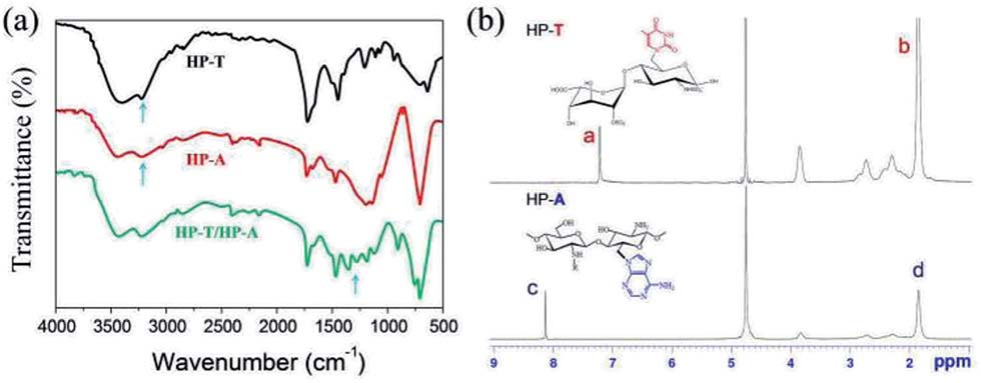
(a) FT-IR spectra of thymine functionalized heparin (HP-T), adenine functionalized heparin (HP-A) and formed hydrogel. (b) ^1^H NMR spectra of HP-T and HP-A.

The successful modification of HP-A and HP-T was confirmed by FT-IR and ^1^H NMR measurement. The FT-IR spectra of the biopolymers are shown in Fig. 2a. The decrease of the band at 3245 cm^−1^ in HP-T indicates that it undergoes a condensation reaction with the primary –OH group of heparin, and the appearance of a new band at 1342 cm^−1^ is due to the C–N stretching vibration between heparin and thymine. In the spectrum of HP-A, a characteristic band at 3254 cm^−1^ and a transmittance peak at 2975 cm^−1^ can be assigned to the –NH stretching of adenine and heparin, respectively. The appearance of a new peak was at 1115 cm^−1^ in HP-A, which corresponds to the C–N stretching vibration, indicating the functionalization of heparin by adenine. The FT-IR spectrum of HP-A/HP-T hydrogels was obtained with additional absorption peaks at 580 cm^−1^ that could be assigned to H–O bonds of scaffold. The functionalization was also confirmed by ^1^H NMR (Fig. 2b). As shown in spectrum of HP-T, the peak at 7.21 ppm is due to the –CH protons of thymine, and the peak at 1.68 ppm corresponds to the –CH_3_ protons of thymine. In spectrum of HP-A, the peaks at 8.12 and 1.85 ppm are assigned to –CH and –NH_2_ protons of adenine, respectively.

**Fig. 3:**
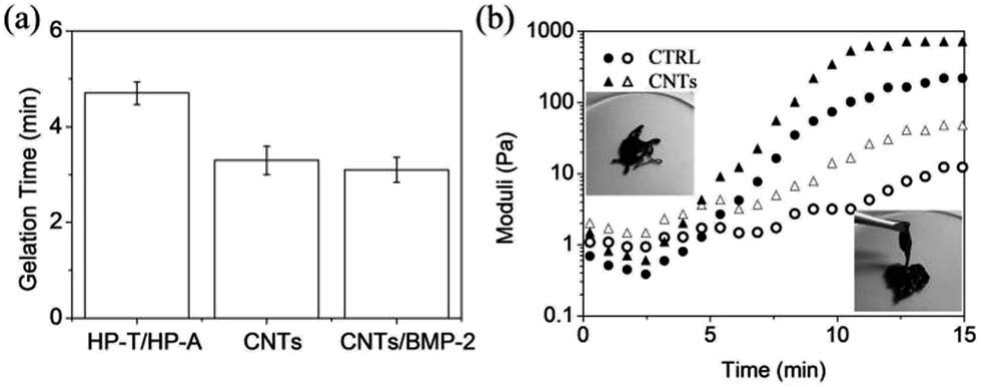
(a) Gelation time of control HP-T/HP-A (CTRL), CNTs and CNTs/BMP-2 scaffolds. (b) Gelation characteristics of CTRL and CNTs as determined by *in situ* rheometry at 37 °C. Occurrence of a gel point as crossover between *G*′ and *G*′′. *G*′, solid symbols; *G*′′, open symbols. Concentration of BMP-2 was 50 ng mL^−1^ of gel scaffolds. Values reported are an average *n* = 5, ±standard deviation.

The gelation can be controlled by the concentration of biopolymers, allowing us to fine-tune the kinetics to an application of interest. Gel scaffolds were formed *via* the mixing of homogeneous solutions of CNT-HP-A and CNT-HP-T in PBS. For purpose of targeted GFs delivery, BMP-2 bonded CNT gel scaffold was prepared using the same conditions, which showed an encapsulation of 50 ng of BMP-2 per mL of gels (referred as CNTs/BMP-2 gel). Higher concentration of biopolymers mixtures was pipetted to ensure homogeneity and immediately resulted in the formation of viscoelastic gel scaffolds. For example, upon mixing with 3.0 wt% solutions of CNT-HP-A and CNT-HP-T, the biopolymers led to self-supporting gel scaffolds within ∼4 min (Fig. 3a), which was significantly faster than the control HP-T/HP-A hydrogel (CTRL) without CNTs (*p* < 0.05). Such rapid curing kinetics is suitable for *in situ* tissue engineering applications. The BMP-2 integration didn't bring significant effect on gelation of the CNTs scaffold (*p* > 0.05).

Dynamic time sweep rheological experiment was conducted to monitor gel network evolution during the gelation process *in situ* at 37 °C (Fig. 3b). The CNT-HP-A/CNT-HP-T mixture displayed rheological behavior that is consistent with formation of a dynamic network. Macroscopic hydrogels were obtained within less than 5 min at which the storage modulus (*G*′) was equal to the loss modulus (*G*′′). The data also indicate a final *G*′ value of ∼100 Pa at time of ∼14 min, signifying a structurally soft network that maintains its 3D shape with loading. The CNTs scaffold is significantly stiffer and stronger than the CTRL without CNTs (*p* < 0.05). Increasing the concentration results in increased scaffold moduli by varying 1.0 to 3.0 wt%, which due to an increase in crosslink density. The insets in Fig. 3b demonstrate the sol–gel transition of the CNTs scaffold. Addition of CNTs does not alter the reversible/irreversible nature of the hydrogel studied, as observed by the constant angular frequency at which *G*′ and *G*′′ cross over, because the lifetime of the cross-link is unchanged. The CNT gel demonstrate the same crossover frequency as the CTRL without CNTs, even though *G*′ plateaus at nearly 500 Pa with 3.0 wt% CNTs, thereby demonstrating a relatively rigid gel network with similar dynamic cross-link properties. Such rheological behavior is characteristic of soft biomaterial, hence it is suitable for injection as a cell scaffold.

### Scaffold characterization

**Fig. 4:**
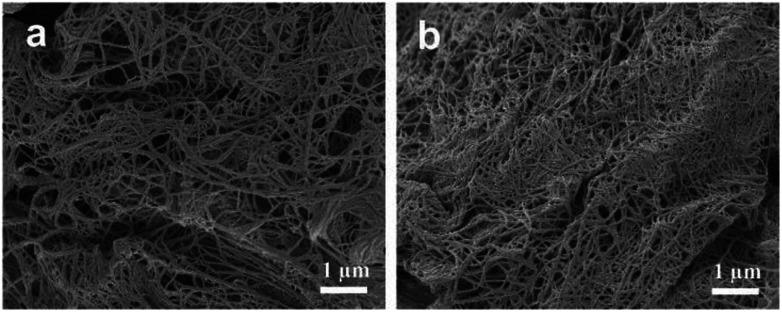
SEM images characterized morphologies of (a) CNTs and (b) CNTs/BMP-2 scaffolds. Concentration of BMP-2 was 50 ng mL^−1^ of gel scaffolds.

Scanning electron microscope (SEM) images characterized the morphologies of CNT gel scaffolds (Fig. 4). According to cross-sectional images (Fig. 4a), the CNT gel scaffold without BMP-2 displayed a nano-structured fibers. The BMP-2 bonded CNT gel scaffold was also observed under the same condition, which showed a similar morphology (Fig. 4b). Therefore, additional BMP-2 didn't significantly affect the morphologies of CNT gel scaffolds. These nano-fibrous architectures of gel scaffolds were attributed to the nature of CNTs, resembling self-assembly nano-fibers. The nano-fibrous CNTs/BMP-2 displayed some nano-particles than the control gel, which might contribute to conjugation of BMP-2 with heparin through the affinity interaction. The number-averaged size of nano-fibers was simultaneously determined by SEM. Size of these nano-fibers ranges from ∼40 nm to ∼120 nm, and the calculated average diameter was 81.3 nm.

**Fig. 5:**
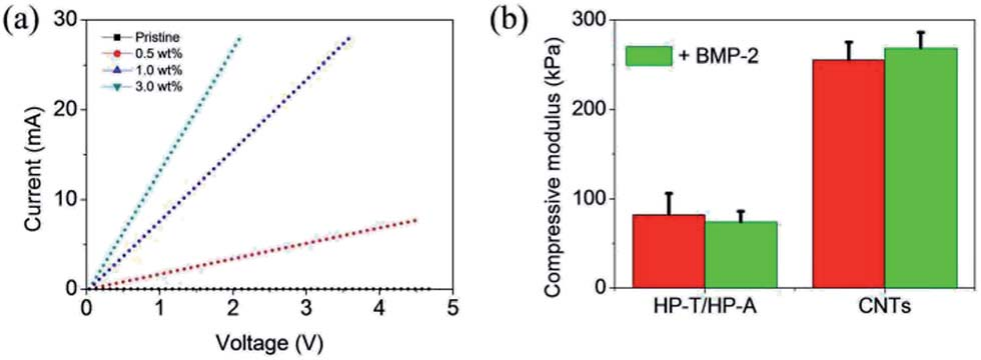
(a) Current–voltage measurements of control and CNT gels as a function of concentrations. (b) Compressive modulus of control and CNTs with or without BMP-2 encapsulation at 37 °C. Values reported are an average *n* = 5, ±standard deviation.

The electrical conductance between the electrodes under the scaffolds was quantified to ensure accurate and reproducible conductivity measurements of the scaffolds. We observed a concentration dependent in the electrical conductivity of CNTs and CNTs/BMP-2 scaffolds. Fig. 5a shows the results of the electrical conductivity measurements at different CNTs concentration. In comparison with pristine heparin scaffold (without CNTs), the electrical conductivity was significantly enhanced upon the addition of CNTs to the scaffolds. Moreover, the scaffolds containing more CNTs had a higher conductivity compared with these low CNTs samples. For instance, the measured currents at an applied voltage of 3 V for the scaffolds containing the 1.0 wt% and 0.5 wt% CNTs were 22.9 mA and 5.1 mA, respectively. However, the conductivity values for CNT gels did not show a significant difference except in 3.0 wt% CNTs concentration in which a sudden increase in the conductivity was occurred.

Compressive modulus of the CNTs and CNTs/BMP-2 gel scaffolds was studied by a dynamic mechanical analysis method (Fig. 5b). The CNTs scaffold had significantly larger compressive modulus than the control HP-A/HP-T gels (*p* < 0.05), which were 256 and 83 kPa, respectively. With addition of BMP-2 in scaffolds, the compressive modulus of the scaffolds was changed correspondingly, whereas no difference was found between them (*p* > 0.05). The data indicated a final modulus value of ∼264 kPa after an addition of 50 ng mL^−1^ BMP-2 in the CNTs scaffold, signifying a structurally robust gel network that maintains its 3D shape with loading.

**Fig. 6:**
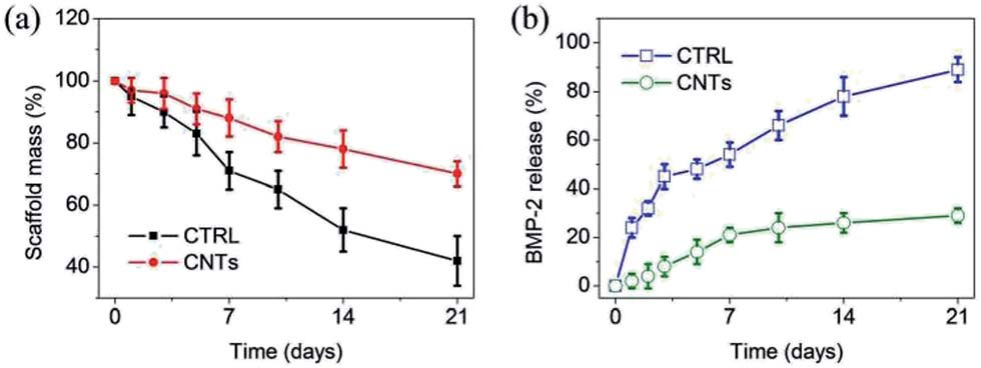
(a) Degradation of control and CNT gels with respect to weight loss in PBS at 37 °C. (b) Cumulative release profiles of BMP-2 from control and CNT gels as a function of time in PBS at 37 °C. Concentration of BMP-2 was 50 ng mL^−1^ of gel scaffolds. Values reported are an average *n* = 5, ±standard deviation.

Biodegradation of scaffolds is critical during bone regeneration, as it enables ASCs to invade the matrix and is also the basis for subsequent remodeling and morphogenesis to form functional bone. Therefore, weight loss of gel scaffolds was monitored as a function of incubation time in PBS at 37 °C. As shown in Fig. 6a, both of the CTRL and CNTs lost their weight steadily up to 21 days. At day 14, the weight remaining ratio of the CTRL and CNTs scaffolds were 54% and 81%, respectively. These results clearly demonstrate that the CNTs served as additional reinforcement in assembled gel scaffolds. This nano-structure influenced many of the macroscopic properties of gel scaffolds, which resulted in a decrease in the mass weight loss. It should mention that the incorporation of BMP-2 also didn't bring a significant influence on weight loss of both gel scaffolds.

Controlled release of BMP-2 can be achieved through their anchorage at predetermined locales of heparin in scaffolds. Strikingly, over a initial period of 21 days, less than 25% of encapsulated BMP-2 was released from the CNT gel scaffold, which showed significantly slower rate compared to the control group (*p* < 0.05) (Fig. 6b). We speculate that this delivery system relied on the active inclusion of heparin in CNT gel scaffold *via* affinity interaction with BMP-2, as well as the stable nano-structure. The BMP-2 slowly diffused out of the CNT gel scaffold, indicating that it was capable of prolonged delivery.

### *In vitro* osteogenesis

It is envisioned that our gel scaffold would not only house stem cells, but also provide osteo-inductive cues in a dynamic functional system that enables cell-induced rearrangement of collagenous matrix and its mineralization. Through this, a microenvironment is enabled to sustain and accommodate osteoblastic differentiation of ASCs homogenously seeded within the scaffolds. To test the hypothesis, human ASCs were seeded within CNTs scaffolds containing BMP-2, and their osteogenesis differentiation and mediated mineralization were compared to those seeded in gel scaffolds alone. The availability of gel scaffolds was allowed to verify that the electrically stimulus (ES) have beneficial effects on the osteoblast proliferation and differentiation.

**Fig. 7:**
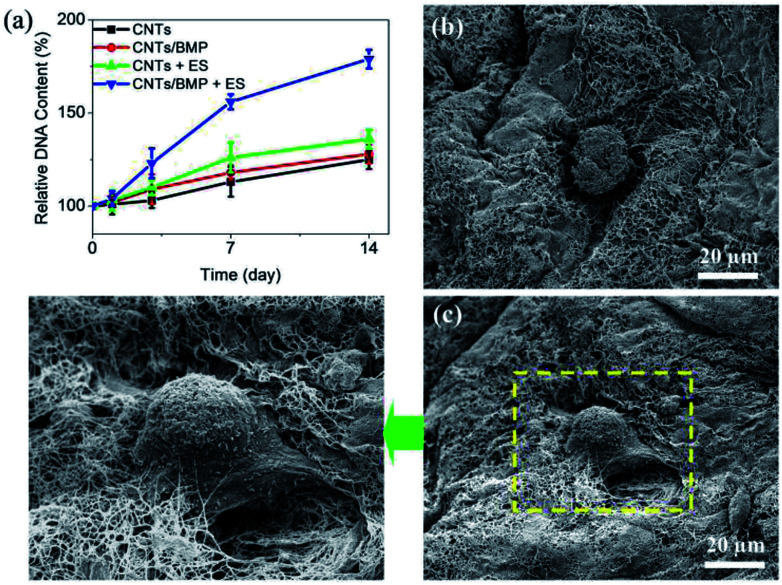
Proliferation of ASCs in scaffolds by electrically stimulus (ES). (a) DNA contents of encapsulated ASCs in CNTs scaffolds as a function of culture time. (b and c) SEM images showed ASCs and BMP-2 containing CNTs matrices after 7 and 14 days of culture. Cell seeding density: 5 × 10^6^ cell per mL gel. Values reported are an average *n* = 5, ±standard deviation.

For bone tissue engineering applications, biomaterials scaffolds often used for loading cells, and to enhance osteogenesis of tissue engineered bone. The transplantation of BMP-2 in biomaterial scaffolds has been studied, since scaffolds with BMP-2 could result in the increase of osteogenic response. Fig. 7a shows the time course of change in relative DNA content of the cells in the CNTs and CNTs/BMP-2 scaffolds. Basically, DNA content in the scaffolds progressively increased after encapsulation compared with initial DNA content, which was further significantly increased after 3 days of culture (*p* < 0.05). As for the ES on cells, there was no significant change of DNA number in the initial 2 days of culture (*p* > 0.05), but its DNA content was significantly increased after 3 days culture (*p* < 0.05). The ASCs residing in the CNTs/BMP-2 scaffolds were observed by SEM after 7 and 14 days incubation (Fig. 7b and c). Roundly shaped cells were distributed in the scaffold after 7 days, indicative of the highly bioactive nature of the CNTs (Fig. 7b). Long incubation time was performed in order to probe a range of differentiative responses of the scaffold for ASCs. As shown in Fig. 7c, after 14 days incubation, the encapsulated ASCs in CNTs/BMP-2 showed polygonous morphologies compare to those on day 7. As the importance evidence for ASCs differentiation, SEM image also showed that lots of collagen-like fibrils accumulated on the cells. These results demonstrated that the multifunctional CNTs/BMP-2 was bioactive and preserved the proliferation and differentiation of the encapsulated ASCs, especially by the ES. The electrically stimulus mediated BMP-2 loaded scaffold induced differentiation of ASCs, and coupled with the cell proliferation data (Fig. 7a), suggested a novel potential mechanism for targeted osteogenesis of stem cells.

**Fig. 8:**
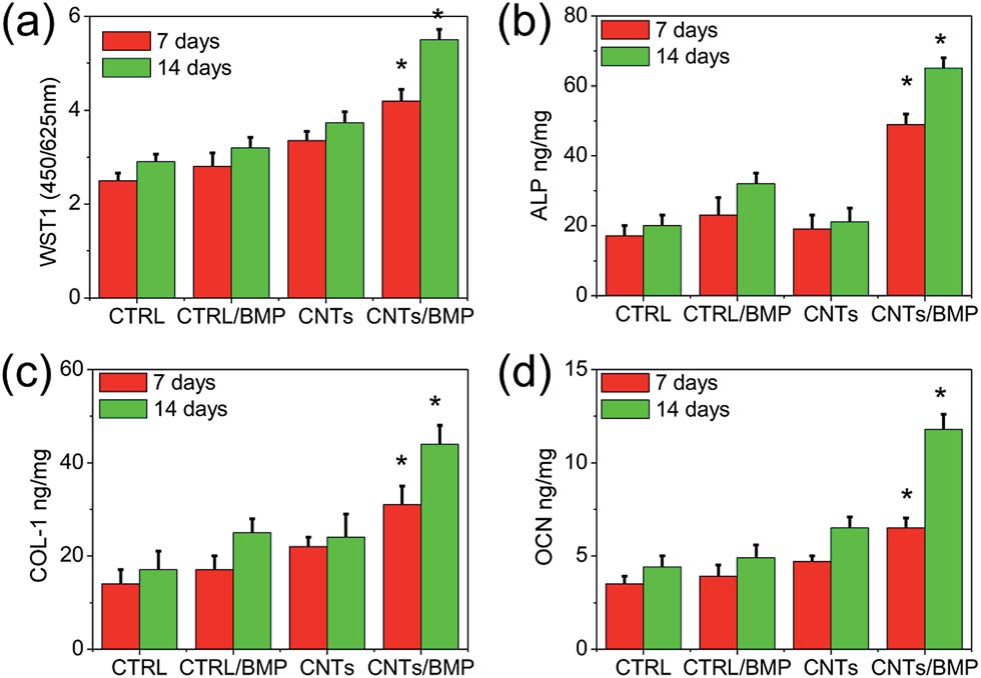
ASCs viability (WST1) and activities (ALP, COL-1, OCN) after 7 and 14 days of electrical culture in control and CNTs scaffolds. Concentration of BMP-2 was 50 ng mL^−1^ of gel scaffolds. Cell seeding density: 5 × 10^6^ cell per mL gel. Values reported are an average *n* = 5, ±standard deviation. Statistical significance is denoted by * (*p* ≤ 0.05).

Recently, CNTs and graphene have attracted significant attention in myocardial tissue engineering applications arising from their outstanding electrical and mechanical properties.^5^ Incorporating CNTs and graphene into cell scaffolds leads to enhanced scaffold flexibility, strength, and electrical conductivity. Although some electrical scaffolds based on CNTs and graphene are quite successful for myocardial tissue engineering, the biomedical applications of these scaffolds are strongly limited by the intrinsic inability to translate ASCs into bone tissue engineering applications. There is a clinical need for development of bioactive gel scaffold that approximate the osteogenesis of ASCs for bone regeneration. In our study, ASCs were cultured up to 14 days by considering the mean time required for the detection of *in vitro* osteoblastic markers production. The BMP-2 deeply influenced cultured cells in gel scaffolds, affecting osteoblast activities. As shown in Fig. 8a, cells grown on BMP-2 containing scaffolds showed an increased metabolic activity. In comparison to controls, BMP-2 and electrically stimulus enhanced Alkaline Phosphatase (ALP) production (*p* < 0.05) (Fig. 8b), which is an early marker of differentiation. In particular, the higher values of type I Pro-Collagen (COL-1) and Osteocalcin (OCN) observed on CNTs/BMP-2 scaffolds at both 7 and 14 days (Fig. 8c and d), indicated a significant stimulation of cell to an early differentiation and deposition of ECM by the electrically stimulus.

**Fig. 9:**
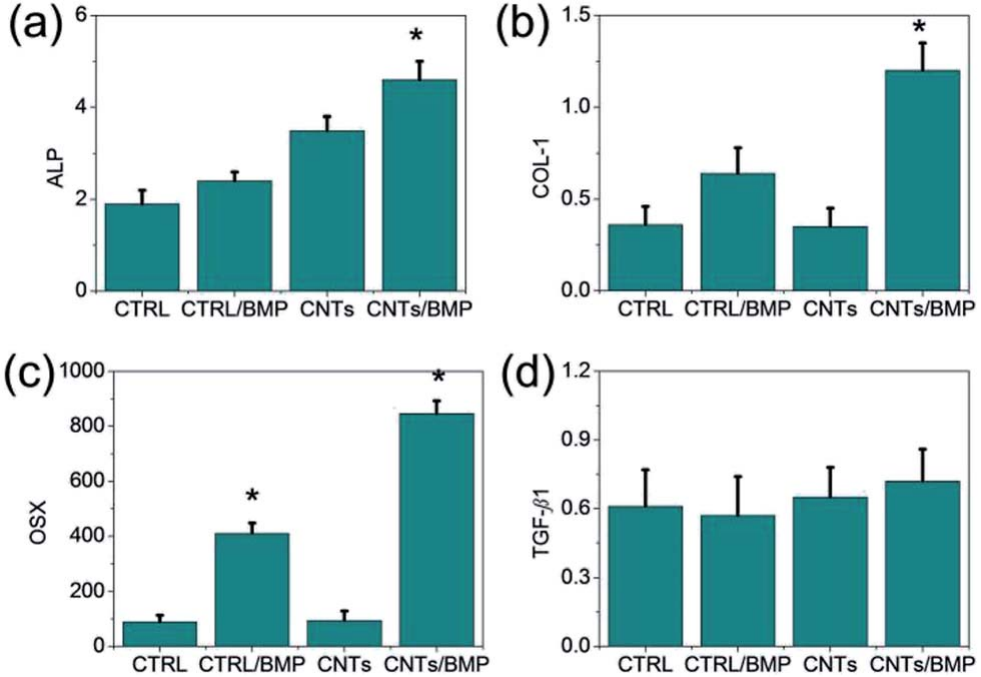
Gene expression of cell growth and osteoblastic phenotype related genes (ALP, COL-1, OSX and TGF-*β*1) during *in vitro* culture after 14 days of electrical culture. Expression level was normalized with respect to the control on day 14. Cell seeding density: 5 × 10^6^ mL^−1^ gel. Statistical significance is denoted by * (*p* ≤ 0.05).

Qualitatively, a higher mineral content was observable in ASCs seeded the CNTs and CNTs/BMP-2 scaffolds after ES incubation, when compared to the control gel. The promotion of CNTs mediated ES to ASCs osteogenic differentiation was assessed by quantitative polymerase chain reaction (qPCR). Four osteogenic differentiation markers at the RNA level were measured. ALP can regulate numerous genes associated with osteogenic differentiation as a key transcription factor in early period, and usually is treated as landmark gene. COL-1 and Osterix (OSX) are the most important two types of representation form in osteogenic differentiation maturation period, and have important effect on osteogenesis. The results of qPCR analysis were in agreement with the significant activation of ALP, COL-1 and OSX expression (Fig. 9a–c). OSX is a gene that specifically regulates osteoblast maturation and differentiation, while transforming growth factor *β*1 (TGF-*β*1) is another important regulator in the bone remodeling network, modulating the interplay between osteoblast and osteoclast in the bone formation-resorption balance. Our results showed that the effects of nano-structures and BMP-2 on cell didn't significantly affect TGF-*β*1 expression on day 14 incubation, as well as the ES culture (Fig. 9d). In comparison with control heparin scaffolds, the CNTs and CNTs/BMP-2 didn't show higher TGF-*β*1 gene expression after 14 days culture, as well as low levels of TGF-*β*1 activity in the culture supernatants. The data on cell activities are consistent with these results, since they show that low levels of TGF-*β*1 correspond to reduced proliferation rates from day 7 to day 14 (Fig. 7a), related to enhancement of osteoblastic differentiation. These results demonstrated that the ASCs were similar to BMSCs in that they were capable of maturing toward bone, which showed similar surface protein marker expression. In combination with electrical stimulate, this electrophysiological CNTs scaffold containing BMP-2 exhibited beneficial effects on ASCs activities and osteogenesis differentiation, which suggested a promising future for bone regeneration.

## Conclusions

We have developed a novelly electrophysiological CNT gel scaffold that specifically allows for targeted BMP-2 delivery and osteogenesis of ASCs. The CNT gel scaffold was attributed to the mechanism of the Watson–Crick base pairing *via* intermolecular hydrogen bonding between thymine and adenine. We demonstrated that the addition of CNTs into biopolymer led to a hybrid scaffold with significantly altered electrophysiological and mechanical properties. Compression modulus and electrical conductivity of the scaffolds, as well as key indicators of their biocompatibility, showed clear dependence on CNTs concentration. CNTs formed electrically conductive and collagen fibril-like nano-fibers, which mechanically strengthened the scaffold, promoted ASCs proliferation and improved osteogenesis. In combination with electrical stimulate, scaffolds fabricated by seeding ASCs onto CNT gels showed strong spontaneous and stimulated synchronous osteogenesis. This electrophysiological gel scaffolds could be used for stem cells to create bone constructs with improved organization, electroactivity and mechanical integrity.

## Conflicts of interest

There are no conflicts to declare.

## Supplementary Material

## References

[cit1] Morishita M., Lowman A. M., Takayama K., Nagai T., Peppas N. A. (2002). J. Controlled Release.

[cit2] Yi D. K., Nanda S. S., Kim K., Selvan S. T. (2017). J. Mater. Chem. B.

[cit3] Bourne D. A., Thomas R. D., Bliley J., Haas G., Wyse A., Donnenberg A., Donnenberg V. S., Chow I., Cooper R., Coleman S., Marra K., Pasquina P. F., Rubin J. P. (2018). Plast. Reconstr. Surg..

[cit4] Khan M. K., Luo J., Wang Z., Khan R., Chen X., Wan Y. (2018). J. Mater. Chem. B.

[cit5] Spinato C., de Garibay A. P. R., Kierkowicz M., Pach E., Martincic M., Klippstein R., Bourgognon M., Wang J. T., Ménard-Moyon C., Al-Jamal K. T., Ballesteros B., Tobias G., Bianco A. (2016). Nanoscale.

[cit6] Song Y., Zhou Y., Chen L. (2012). J. Mater. Chem..

[cit7] Fan M., Yan J., Tan H., Miao Y., Hu X. (2014). J. Mater. Chem. B.

[cit8] Tan H., Wu J., Huang D., Gao C. (2010). Macromol. Biosci..

[cit9] Tan H., Ramirez C. M., Miljkovic N., Li H., Rubin J. P., Marra K. G. (2009). Biomaterials.

[cit10] Xu X., Jha A. K., Duncan R. L., Jia X. (2011). Acta Biomater..

